# Chances of Life

**Published:** 1871-09

**Authors:** 


### CHANCES OF LIFE.

If a child lives twelve months, the chances are equal it will reach thirty-three years. From ten to fifteen, life is most secure; one who reaches that age will most probably live to be fifty-five years old.

A mother never forgets, never "gets over" the death of her first-born in infancy; and when it is taken into account that the young mother is almost wholly ignorant of everything pertaining to the management of infants, whose lives are placed in the hands of the monthly nurse with all the antiquated ideas of a past age, it is no wonder that there are so many Rachels in the land.

The most vitally important points in the care of infants are, 1st. Keep them abundantly warm all the time, then they can never take cold; not the warmth of overheated rooms, but the warmth connected with appropriate clothing; woolen next the skin always. 2d. Regular eating; it may be safely said that more than half of all the infants who die have been destroyed by injudicious feeding. 3d. Perfect cleanliness of person. 4th. Daily opportunities of breathing out-door air, except when damp or cold.

